# Extracellular Vesicles in Neurodegenerative Diseases: An Update

**DOI:** 10.3390/ijms241713161

**Published:** 2023-08-24

**Authors:** Smara Sigdel, Sabrina Swenson, Jinju Wang

**Affiliations:** Department of Biomedical Sciences, Joan C. Edwards School of Medicine, Marshall University, Huntington, WV 25755, USA; sigdels@marshall.edu (S.S.); swenson4@marshall.edu (S.S.)

**Keywords:** extracellular vesicles, neurodegenerative disease, dementia, mild cognitive impairment, Alzheimer’s disease, Parkinson’s disease

## Abstract

Neurodegenerative diseases affect millions of people worldwide. The likelihood of developing a neurodegenerative disease rises dramatically as life expectancy increases. Although it has drawn significant attention, there is still a lack of proper effective treatments for neurodegenerative disease because the mechanisms of its development and progression are largely unknown. Extracellular vesicles (EVs) are small bi-lipid layer-enclosed nanosized particles in tissues and biological fluids. EVs are emerging as novel intercellular messengers and regulate a series of biological responses. Increasing evidence suggests that EVs are involved in the pathogenesis of neurodegenerative disorders. In this review, we summarize the recent findings of EVs in neurodegenerative diseases and bring up the limitations in the field.

## 1. Introduction

Neurodegenerative diseases are disorders featuring progressive neuron loss in the central and peripheral nervous systems, creating issues in movements and mental functions [1]. Common neurodegenerative disorders include Alzheimer’s disease (AD), Parkinson’s disease (PD), mild cognitive impairment (MCI), frontotemporal lobar degeneration, dementia with Lewy bodies, and dementia [2]. During the past decades, great efforts have been undertaken to understand the mechanisms of neuronal cell death in neurodegenerative diseases [2]. Several mechanisms such as oxidative and nitrosative stress are shown to be some of the reasons for neuronal cell injury, which could trigger the aggregation of misfolded proteins in the central nervous system and cause mitochondrial dysfunctions and neuro-inflammations and eventually results in neurodegenerative disorders [3], but the entire molecular mechanism is still not completely understood.

Extracellular vesicles (EVs) are lipid bilayer structures formed and released by virtually all live cells. EVs contain bioactive molecules such as proteins, non-coding RNAs, and lipids. They are emerging as a novel form of message exchange via conveying cargo to target cells. EVs are believed to be one of the most powerful tools for intercellular communication in both physiological and pathological processes [4,5,6]. The diverse cellular processes that EVs are involved in make them a promising source for both diagnostic and therapeutic applications. Recently, altered cargo in EVs has been identified in patients with neurodegenerative diseases, indicating their potential application as a biomarker. In this study, we discuss the novel findings of EVs in neurodegenerative diseases and bring up the limitations hindering the potential broad application of EVs in the clinic.

## 2. The Biogenesis, Contents, and Imaging of EVs

### 2.1. Biogenesis of EVs

**Figure 1:**
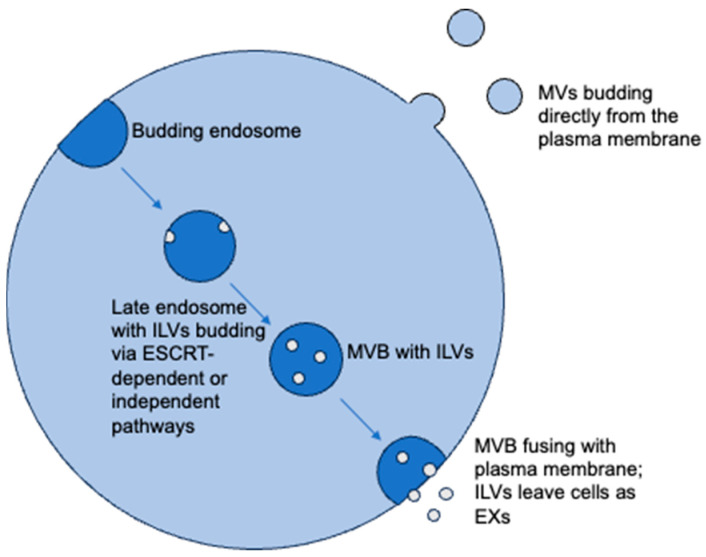
EXs and MVs are generated via differing pathways. While EXs require endosomes for biogenesis, MVs bud directly from the plasma membrane. EX generation may occur via ESCRT-dependent or ESCRT-independent pathways, although when and why each one is utilized has not been completely defined.

The two primary types of EVs, exosomes (EXs) and microvesicles (MVs), are classified on the basis of biogenesis [7]. EXs, the smallest EVs ranging from 10 to 150 nm in diameter, were first observed by Pan and colleagues by immunoelectron microscopy in 1985. EX biogenesis occurs under normal conditions but can be further stimulated by innumerable conditions [8]. In general, the biogenesis of EXs (Figure 1) begins when the cell membrane buds in the intracellular direction to form endosomes [9]. Within the endosomal system, cargoes are sorted into early endosomes, which then mature into late endosomes or multivesicular bodies. Late endosomes are specialized endosomal compartments that are rich in intraluminal vesicles (ILVs). The ILV budding is facilitated by the endosomal sorting complexes required for the transport machinery (ESCRT) pathway and machinery via its four complexes, ESCRT-0 to ESCRT-III [10]. ESCRT-0 utilizes hepatocyte growth-factor-regulated tyrosine kinase substrate to initiate the process by attaching ubiquitinated cargo; ESCRT-I is summoned via tumor susceptibility gene 101 (TSG101) to begin budding of ILVs when ESCRT-II binds; ESCRT-III divisions Vps20, Snf7, Vps2, and Vps24 continue to encourage ILV formation; and then Vps4 is used by ESCRT-III to cleave the ILVs from the endosomal membrane [11,12,13,14]. After the ILV formation is complete, the endosomes have effectively become multivesicular bodies (MVB), which can bind with the plasma membrane, causing the ILVs to leave the cell as EXs [15]. MVBs can also fuse with lysosomes, inducing ILV degradation and no production of EXs [16].

It is important to note, however, that there are also ESCRT-independent pathways that result in EX formation, although more complex and significantly less common [17,18]. Trajkovic and colleagues found that ceramide can trigger EX biogenesis in oligodendrocytes [17]. In addition, there are various other EX generation pathways that are ESCRT- and ceramide-independent [12], but it is still unclear whether the sorting and sequestering of molecules involve different mechanisms, and consequently proteins, or if there are various MVB subpopulations within singular cells. Some studies suggest that EXs may also be produced directly by the plasma membrane, although it is more widely accepted that EXs mostly arise from MVBs [19].

MVs are slightly larger than EXs with a size range between 100 nm to 1000 nm in diameter, and they are unique in that they form directly from the extracellular budding of the plasma membrane [20]. The biogenesis of MVs is less studied than that of EXs but thus far has been shown to be dependent on the transfer of plasma membrane phospholipids from the internal to outer leaflet, followed by the increased rigidity of the membrane via actin–myosin interactions [21]. Simply, any alteration in regulated plasma membrane asymmetry provides a route for MV synthesis, including protein-induced membrane flexibility by enzymes such as sphingomyelinases in the interactions with sphingomyelin and intramembranous cholesterol [22]. The primary mechanism of MV release then depends on cytoskeletal alterations by calpain, a protein activated by the influx or endoplasmic reticulum production of calcium (Ca^2+^) [23,24]. The Ca^2+^ concentration can also be involved in altering the structure of the plasma membrane, meaning that Ca^2+^ can alone induce both MV synthesis and release [25]. Ceramide is also involved in MV production, either by sphingomyelin conversion to ceramide by sphingomyelinases or just by being introduced to the plasma membrane and interrupting the normal curvature [26].

In addition, the extent of EV release could be influenced by a variety of factors, including cellular status, the microenvironment, PH, and intracellular calcium levels, etc. Hypoxia is a physiological state that influences EV cargo and release in multiple cell types. It has been shown that EXs released under hypoxic conditions carry factors that are correlated with increased tumor invasion and metastasis [27]. EXs derived from hypoxia-preconditioned mesenchymal stromal cells were shown to rescue cognitive impairment in the Alzheimer APP/PS1 mouse model [28], while ischemic preconditioned astrocyte-derived EXs can protect neurons from apoptosis [29]. Our group has demonstrated that other conditions, such as inflammatory and starvation stimuli, can differently affect the functions of MVs released by endothelial progenitor cells [30]. Additionally, we have observed that interventions such as exercise can alter the secretion and cargo of EVs from bone marrow-derived endothelial progenitor cells [31]. Others found that PH can influence the number of EXs released from tumor cells and Hela cells and increase the loading of nucleic acids in EXs derived from HEK293 cells [32,33,34]. Increasing levels of intracellular calcium have also been shown to increase MV shedding from tumor cells and EX release from oligodendrocytes and platelets [35,36,37]. Calcium ionophore-induced EV release exhibited protective effects against reperfusion injury in HEK293 cells [38,39]. In addition, Rab proteins, a subfamily of small GTPases, can influence both EX and MV release and cargo packages [39]. Decreasing the expression of Rab22a in a human neuroblastoma cell interferes with the accumulation of amyloid beta protein in EXs under hypoxic conditions [40].

Because EVs adopt the characteristics of parent cells, they could be complicated in the pathogenesis/development of diseases by carrying pathogenic proteins or other biological materials [41]. Strategies to inhibit EV biogenesis may be an alternative way to alleviate disease occurrence or progression. Catalano and colleagues have extensively reviewed pharmacological treatments that can be used to minimize disease spread by inhibiting EV production, including GW4869 for EXs and Y27632 for MVs [26]. More recently, Kim and colleagues have also listed several other compounds that also affect coenzyme-a, phospholipids, calcium, and cytoskeletal elements [42].

Taken together, EX and MV biogenesis is a finely tuned and reactive pathway with multiple molecular players that are involved in other critical cellular functions or vesicle-related physiologic and pathologic roles.

### 2.2. Contents of EVs

The composition of EVs varies based on cellular origin and biogenesis. Generally, EVs carry proteins, lipids, and nucleic acids. Some EX compositions are thought to be ubiquitous regardless of parent cells. There is a database, Exocarta, highlighting the vast number of proteins, lipids, and nucleic acids present in EXs from different cell types [43].

Those proteins involved in EV biogenesis, such as Alix and Tsg101 (a protein in the ESCRT-I complex), are considered ubiquitously expressed proteins and are used as general markers of EXs [44]. Other constitutive proteins of EXs are tetraspanins, which are a group of membrane proteins that have been shown to influence cargo trafficking in fibroblasts [45,46]. Tetraspanin CD63, CD9, and CD81 are also used as specific exosomal markers [47,48,49]. Further, heat shock proteins are commonly found in EXs due to their participation in protein homeostasis [50,51]. Other exosomal proteins include nSMase 2 of the ceramide-dependent pathway [52,53,54], actin and flotillin, SNARE, and Rab [55]. Interestingly, EXs have not been shown to carry proteins associated with endoplasmic reticulum or Golgi apparatus origins [56], while MVs have proteins with higher levels of post-translational modifications [57,58].

Lipids play an essential role in maintaining EX rigidity, membrane excretion and internalization, and intravesicular function of EXs [59]. Lipid rafts also play roles in sorting lipids into EX membranes [60]. EX membranes have different lipid composition profiles based on the parent cell type but generally feature cholesterol, sphingomyelin, glycosphingolipids, and phosphatidylserine [61,62]. Cholesterol is involved in the localization of late endosomes as well as the secretion of EXs and MVs [63]. This, of course, has been shown to be cell-type dependent [64]. Lipids have also been shown to be involved with the internalization process of recipient cells [59].

While some findings show that genomic and mitochondrial DNA is found within EVs, microRNAs (miRs) are shown to be one of the most abundant RNA types in EXs [65,66]. Other types of RNAs present include messenger RNA, ribosomal RNA, long non-coding RNA, piwi-interacting RNA, small nuclear RNA, and small nucleolar RNA [55]. Many RNAs have been shown to be involved in a variety of biological processes including neurogenesis in the progression of neurodegenerative diseases such as PD and AD [67,68,69,70]. For example, EXs released from hypoxia-preconditioned mesenchymal stromal cells can improve the cognitive function of APP/PS1 mice by rescuing synaptic dysfunction and regulating inflammatory responses [28]. Hou and colleagues have demonstrated that exercise-intervened EXs carrying miR-342-5p could exhibit cardioprotective effects [71]. All these findings indicate that EVs contain various bioactive molecules and convey these cargoes to recipient cells to alter cell function. To date, little is known about how these bioactive molecules are loaded into EVs. Some studies suggest that passive and selective packaging, as well as CD63-mediated sorting, assist with cargo loading [72,73], but more studies are needed to advance our knowledge in this respect.

### 2.3. Imaging of EVs

Several EV labeling techniques have been developed for tracing EVs and advancing their function study in vivo, including fluorescent labeling, radioisotope labeling, cell-engineering techniques, etc. Hence, the biodistribution of EVs can be monitored in vivo using a variety of imaging modalities, including positron emission tomography (PET) [74], magnetic resonance imaging (MRI) [75], and bioluminescence imaging [76].

Lipophilic fluorescent dyes such as DiO, DiI, DiD, DiR, Pkh26, and Pkh67 are often used to stain the membranes and other lipid-soluble biological structures of EVs [77,78,79]. Wen and colleagues labeled mesenchymal stem-cell-derived EVs with DiD to achieve in vivo imaging of EVs at radiation-damaged sites in mice [80]. Others have applied Pkh 26 or Pkh67 to label EVs and achieved in vivo tracing of the injected EVs in murine models [81,82]. Thiol-reactive dye such as Alexa488 is also used to label EVs by combining fluorescent groups with maleimide. Using this dye, Roberts-Dalton et al. reported that EVs can be incorporated into Hela cells in a clathrin-independent endocytosis mechanism [83]. Radioisotope labeling is another classical laboratory labeling technique. Compared with traditional optical labeling techniques, radioactive labeling has high sensitivity and stable imaging performance for in vivo imaging. Iodine and metal isotopes are commonly used for labeling. Royo et al. have demonstrated that the ^124^I-labeled EVs can be detected in the liver and the brain of the recipient mice using PET [84]. However, the use of radioactive substances requires large precision instruments and is under strict surveillance, especially for clinical applications; its applications in EV labeling are relatively few. Recently, using genetic engineering techniques, researchers integrated the coding sequences of fluorescent proteins or luciferases with tetraspanins proteins such as CD63 to construct fusion proteins to label and trace EVs under specific conditions. For instance, an EX-reporter mouse model expressing CD63–GFP fusion protein was used to study the intercellular communication between neurons and astrocytes in the central nervous system [48]. Takahashi et al. established a reporter model of a Lactadherin and Gaussia Luciferase fusion protein, expressed in mouse melanoma B16BL6 cells [85]. In addition, other fluorescent proteins such as EGFP, tdTomato, and mNeonGreen have also been developed as a tool for visualization and uptake studies of EVs [86,87].

## 3. EVs in Neurodegenerative Diseases

### 3.1. The Potential Roles of EVs in Dementia and MCI

Dementia is a syndrome that impacts cognition, resulting in decreased function in work, lifestyle, and social contexts. Dementia may be neurodegenerative or reversible, depending on the disease or injury inducing it. However, it is important to note that most instances of dementia are a result of multiple neurological disorders or injuries. The prevalence of dementia is expected to increase to 75 million cases internationally by the conclusion of the decade, with the rising numbers attributed to aging populations. While most dementias result from protein misfolding and accumulation, vascular dementias are linked to blood loss from cerebral vasculature following brain injuries like strokes [2]. The general development of MCI in individuals is much less defined than dementia and its subtypes; it ranges from being neurodegenerative to not and may not even result in the onset of dementia and AD.

The risk factors of dementia and mild cognitive impairment (MCI) include aging, genetics, cerebral injury, cardiovascular disease, educational attainment, and exposure to toxic materials, although risk factors typically appear in combinations in patients with dementia [88]. At a molecular level, dementia development is hypothesized to be in response to toxic Aβ proteins (Aβ), altered tau proteins, and/or α-synuclein (α-syn) proteins [89,90,91]. These proteins typically arise in one portion of the brain before gradually spreading. The systemic route in which toxic proteins manifest throughout the brain is considered when diagnosing stages of dementias such as Alzheimer’s; however, the mechanisms underlying the pattern of protein aggregation are not clear. The most closely linked yet least understood risk factor contributing to dementia is having two APoE4 alleles, which only appears in a small fraction of individuals. APoE4 often exacerbates other risk factors such as cardiovascular and neurological diseases; the gene and the diseases simultaneously result in the upregulation of toxic proteins and cerebral inflammation [92].

Within the past decade, research has turned to EVs to uncover the mechanistic route of dementia and its comorbidities’ onset and progression (current findings are summarized in Table 1). EVs may have either negative or positive effects depending upon the cells that they originate from and may have roles in the advancement of diseases by dispersing toxic cargoes [93]. It is further established that all cells of the central nervous system, as well as endothelial cells, deposit EVs that participate in the body’s inter-organ communication [94,95]. Current research aims to identify whether EVs secreted by cells within the brain can transmit toxic RNAs, lipids, or proteins and the mechanisms in which this may occur. Increasing evidence has uncovered that EVs carry toxic proteins that contribute to the development of dementias under numerous disease conditions, such as AD and PD [96]. It has been also proposed that EVs such as EXs can transmit miRNA and lipids within the brain, promoting cell death and inflammation, especially during AD and PD-induced dementia [97].

Endothelial cells have been suggested as possible contributors to disease spread and exacerbation due to their respective roles in performing as sites of inflammation and protein accumulation in dementia syndrome [95]. One study has linked cerebral endothelial-cell-derived small extracellular vesicles (CEC-sEVs) to causing cognitive dysfunction in diabetic conditions; CEC-sEVs extracted from healthy mice, as expected, reduced cognitive decline when administered to diabetic mice [98]. Recently, another report revealed that microglia-derived vesicles were found in higher concentrations in frail patients with MCI compared with non-frail ones, pointing to their participation in cognitive decline. These microglia-derived vesicles were also factors in creating neuronal damage. The article concludes by stating that microglia dysfunction is common in various cognitive diseases and expedites the formation and release of Aβ proteins, which may be via EVs [99].

Other forms of dementia, such as vascular dementia, are also studied alongside AD and MCI because of their common appearances as comorbidities. The risk factors for vascular dementia differ slightly; while they still include aging and genetics, a higher significance is placed upon physical, preventable health and lifestyle choices, such as hyperglycemia, hypertension, and obesity. A study examining diabetes as a risk factor for vascular dementia notes that the efflux of proteins such as hsp60 from neuron EXs is elevated in diabetic individuals, resulting in astrocytes taking up the hsp60 and spreading oxidative stress and, hence, inflammation throughout the brain [100]. This inflammation occurs by damaging neurons and glial cells.

Frontotemporal lobar degeneration (FTLD) is another type of dementia, in which neurons in the frontal and temporal lobes of the brain are destroyed by the accumulation of TAR DNA binding protein-43 (TDP-43), resulting in extensive memory and function loss. Those with frontal lobar degeneration-dependent dementia are significantly more likely to feature mutations in the progranulin gene (GRN), and studies have concluded that EVs from neural, astrocytic, and microglial cells can accumulate and spread toxic TDP-43 [101,102]. Clinical studies have found that EXs can transmit TDP-43 in patients with FTLD in a prion-like manner, which then leads to neuronal death [103]. Other groups have indicated that TDP-43 was detected in EXs released from neuroblasts and primary neurons [104].

Outside of disease progression, EVs have the potential to serve as biomarkers. Upadhya et al. have demonstrated that in various neurocognitive diseases, circulating EVs released by astrocytes may play a role in identifying the severity of the condition and, in non-degenerative cases, provide a route to measure the stages of healing [105]. The development of dementia from MCI to dementia can be examined closer using proteomic biomarkers such as hsp1A, puromycin-sensitive aminopeptidase, and prostaglandin F2 receptor negative regulator, as per Muraoka and colleagues [106]. In terms of the Aβ hypothesis, it has also been suggested that plasma EVs can indicate how much MCI has advanced [107]. For FTLD, the TDP-43 levels in astrocyte EVs isolated from plasma can serve as a novel biomarker [102]. EXs in diseased individuals are prone to containing higher concentrations of neurofilament light chain levels, meaning that EXs could be used to anticipate FTLD onset [108]. Sproviero et al. observed distinct differences in mRNA cargoes in EVs sourced from AD, PD, and FTLD samples and those from control samples. These mRNAs are related to serine arginine-rich protein families, kinase genes, splicing, and heat shock proteins [109]. Another study found that EV concentrations increased and the average size decreased in patients with AD, DLB, and FTLD [110]. For dementia with Lewy bodies (DLB), biomarkers may include the RNA expression levels in small EVs circulating in plasma or altered protein levels in plasma EVs, such as gelsolin and butyrylcholinesterase [111,112,113]. EVs in the cerebral spinal fluid of DLB subjects may also be involved in abnormal ceramide concentrations and metabolism [114].

**Table 1:** Findings on the roles of various EVs in the pathogenesis or diagnosis of neurocognitive/dementia-related disorders.

EV Types	Roles	Related Disease	Reference
Microglial MV	Conversion of Aβ from normal to pathogenic forms by lipids	MCI	[115]
Microglial EVs	Induce neuronal damage by increasing genesis and release of Aβ	MCI	[99]
Cerebral endothelial EVs	Prevent neurogenesis and enhance cognitive dysfunction in diabetic conditions	Dementia	[98]
Neuron EXs	Spreading hsp60 proteins to astrocytes	Diabetic-associated vascular dementia	[100]
Circulating EVs in plasma	Biomarker	MCI	[107]
Astrocyte-sourced EVs in plasma and CSF	Biomarker	FTLD	[105,108]
Circulating EVs in plasma/blood/serum and CSF	Biomarker	DLB	[111,112,113,114]

### 3.2. The Potential Roles of EVs in AD

AD is the most common and well-known form of dementia that has been well studied yet still not fully understood. AD coincides with vascular dementia in most cases, indicating a link between AD-induced cognitive decline and cardiovascular disease [88]. Despite having such a high prevalence, no pharmaceutical treatments have been made available in the market for AD. Some have suggested that the lack of medicines for those suffering from AD can be attributed to a failure to address amyloid/tau accumulation simultaneously with cerebrovascular diseases [90].

Previously, it was supposed that contact between cells in the brain was responsible for the progress of AD [116]. In recent years, many laboratories have begun to consider EVs as a potential mode of transportation for toxic biomaterials within the brain [117]. Endothelial cells, for instance, which are important components of the blood–brain barrier (BBB), have been noted to produce EVs containing Aβ proteins [95]. These EVs containing toxic Aβ proteins across the BBB effectively reduce the production of neurons from neuron progenitor cells by inducing mitochondrial dysfunction and oxidative stress. This causes a cyclic pattern in which increasing Aβ levels damage the BBB, forming a dysfunctional influx/efflux of EXs that also contain Aβ, driving neurodegeneration.

The body of research on the spread of AD via EVs generally focuses on EVs of glial or neuronal origin in AD progression [94,115]. This is supported by work that indicates that most EV particles shift from neurons to glial cells and that Aβ and tau protein concentrations in EVs increase as AD develops [118,119]. Muraoka and colleagues reported that brain-derived EVs carry a higher level of signature proteins Aβ and tau and glial-specific molecules such as ANXA5, VGF, GPM6A, and ACTZ in AD patients compared with control subjects [118]. The combination of Aβ, tau, and brain-cell-specific proteins, including ANAX5, VGF, GPM6A, or ACTZ, may serve as potential biomarker candidates in AD patient body fluid samples. Asai et al. also found that reduced microglial cell concentrations and microglial EX production in the brain are related to diminished tau proteins in AD [120]. In inflammatory conditions, microglia can also communicate signals with each other, resulting in lipids found in both microglia intracellularly and released via MVs extracellularly to initiate the conversion of Aβ from normal insoluble forms to toxic soluble versions [115]. Additionally, the spreading of tau via EXs is also shown to have occurred between neurons and microglia [121]. Microglia-derived MVs displayed neurotoxicity to microglia and cortical neurons by trafficking neurotoxic Aβ proteins [115,122]. Some studies have discovered that astrocyte EVs were increased in cerebrospinal fluid in AD relative to MCI patients and controls, further pointing to the idea that glial cells serve as the mode of AD proliferation [106]. Another finding that highlights the potential importance of glial cells in the spread is that because they serve as the primary phagocytic cells within the brain, they have an amplified ability to both phagocytize and excrete vesicles compared with neurons [120]. Nevertheless, this is in direct contrast to studies that propose protein distribution between neurons in a prion-like fashion without glial cells as mediators [123]. When proteins such as tau are released via EXs, they can induce constitutive tau proteins to convert to their toxic forms [124,125]. In studies where neurons distribute EVs to each other, microglia have been observed to serve as a means to degrade EXs rather than releasing them [53].

Both astrocytes and neurons produce EVs with increased tau and Aβ levels in AD conditions [126,127,128]. Therefore, a biomarker for AD could be established by measuring Aβ and tau protein concentrations in EVs isolated from CSF and blood [129,130]. It has also been observed that EXs released from astrocytes may have increased levels of inflammatory proteins that can be utilized as biomarkers [131]. Proteins also isolated from EXs produced by neurons include Aβ and p-tau proteins and have the potential to serve as biomarkers [130,132]. Specifically, increasing levels of toxic proteins in neuron EVs correspond to the progression of AD throughout the brain [133]. mRNA and miRNA are also cargoes found in neuron-derived EXs that could be utilized as biomarkers of cognitive impairment in AD [134]. Outside of astrocytes and neurons, miRNAs, inflammatory factors, and toxic proteins also found in microglia, released EXs can also serve as biomarkers [120,135]. Jia et al. conducted a multicenter study with four independent datasets and found that a panel of miRs was changed in subjects with AD and could detect preclinical AD five to seven years before the onset of cognitive impairment [136]. Markers such as Alix also indicate increased release of EXs around diseased areas of the brain [117]. There is currently an abundance of research being conducted on EVs as novel biomarkers for AD, exploring more specificities of the relationship than with other neurocognitive disorders.

### 3.3. The Potential Roles of EVs in PD

While, like AD, PD can result in dementia and cognitive decline, PD also presents itself with physical challenges like bradykinesia and tremors. This indicates the primary manifestation of the disease is not in the entorhinal cortex or hippocampus but in the substantia nigra responsible for one’s movement [137]. PD also is associated with Aβ, tau, and neuron-damaging α-syn proteins that form Lewy bodies characteristic of PD and LBD [138].

Recent studies have indicated that EVs such as EXs can serve as transporters of α-syn oligomeric proteins throughout neurons in the brain, expediting the spread and growth of the Lewy bodies [139]. These findings have been supported extensively by studies that indicate that neuron-based EXs containing α-syn oligomers are more likely to be taken up by neurons than those without α-syn proteins [140,141]. However, like with AD, it is not universally accepted that the transmission of EVs occurs neuron to neuron, and some have shown that EXs can be released by glial cells before being absorbed by neurons [142]. Secretion of the toxic α-syn proteins can be induced by high intracellular concentrations of calcium, but the pathological pathway has not been completely elucidated [143,144]. In diabetes, the negative impact of EVs on those with PD is increased due to the EX-based transportation of α-syn from the pancreas to neurons [145]. Aside from proteins, research groups have shown that miRNA such as novel miR-44438 in EVs reduces α-syn disposal by neurons, resulting in their buildup within neurons [146]. Other RNAs, such as miR-34a, which are packaged into EXs and secreted by astrocytes, promote neurotoxicity and inflammation in PD by increasing the concentration of toxic molecules and inflammatory proteins [142,147]. Clinical studies reported that specific miRs (hsa-miR-23a-3p, hsa-miR-126-3p, hsa-let-7i-5p, and hsa-miR-151a-3p) significantly decreased in AD with respect to controls [148].

EVs can also serve as biomarkers within the context of PD. Bloomer and colleagues identified neuron EXs isolated from plasma as potential biomarkers because they can feature different levels of α-syn oligomers, Aβ, p-tau, and insulin signaling-related proteins [149]. Further, it has been shown that elevated α-syn levels in neuron EXs found in serum precedes fully developed PD, demonstrating novel ways that they could serve as biomarkers [150]. PD, like AD, also includes altered exosomal cargo profiles, including altered levels of miRs (miR-153, miR-409-3p, miR-10a-5p, and let-7g-3p), mRNAs of the amyloid precursor protein, α-syn, Tau, neurofilament light gene, DJ-1/PARK7, and long non-coding RNAs (RP11-462G22.1 and PCA3) in EXs in cerebral spinal fluid [68], suggesting that cerebral spinal fluid exosomal RNA molecules could serve as biomarkers in regard to specificity and sensitivity in differentiating PD from healthy controls.

All these findings suggest that EVs could be a therapeutic target for PD and AD. Current findings regarding the roles of different sources of EVs in PD and AD are summarized in Table 2.

### 3.4. The Promise of EVs in Treating Neurodegenerative Diseases

EVs can also function as a novel therapeutic tool for neurodegenerative diseases given that they can penetrate through the BBB and target the brain. On a general level, Raghav and colleagues have systematically reviewed sixty articles on how EVs may serve as therapeutics in neurodegenerative diseases; studies have stated that patient-specific or parent-cell-specific EVs can be used to catalyze regeneration post-damage, transport beneficial siRNAs and pharmaceuticals, and restore neurological functions [151]. Alvarez-Erviti et al. engineered EXs to express the central-nervous-system-specific rabies viral glycoprotein peptide and found these EXs exhibited the capability of delivering siRNA to neurons, microglia, and oligodendrocytes, demonstrating a significant reduction of Aβ deposits in the mouse brain [152]. As demonstrated by Yuyama et al., EXs can act as scavengers of extracellular Aβ by trapping the Aβ on surface glycosphingolipids and transporting it into microglia in the AD mouse brain [153]. Xia and colleagues have also conducted a more narrow review of the therapeutic effects of specifically EVs produced by transplanted stem cells, which may reduce Aβ and α-syn deposition, apoptosis, and oxidative stress in addition to promoting angiogenesis and cell regeneration [154]. More specifically, mesenchymal stem-cell-derived EVs can convey specific cargo such as miR-29c-3p to neurons, which then inhibits BACE1 expression while activating the Wnt/β-catenin pathway [155]. EVs released from neural stem cells also exhibit neuroprotective effects, restore fear extinction memory consolidation, and reduce anxiety-related behaviors of the 5xFAD accelerated transgenic mouse model of AD [156]. Cui and colleagues revealed that EXs derived from hypoxia-preconditioned mesenchymal stromal cells can rescue cognitive impairment in the Alzheimer APP/PS1 mouse model [28].

An increasing number of studies are turning to implementing exercise intervened EVs to see if they could be another mechanism through which exercise can produce biological changes in the central nervous system. Fruhbeis et al. reported that exhaustive cycle and treadmill exercise raised EVs secretion into the circulation immediately post-exercise, which returns to baseline after 90 min [157,158]. Our group has demonstrated that moderate treadmill exercise intervention (10 m/min for 60 min for 8 weeks) regulates the functions of bone marrow endothelial progenitor-cell-derived EXs in C57BL/6 mice [31]. Furthermore, our recent study revealed that the exosomal communication between endothelial progenitor cells and brain cells is compromised in hypertensive conditions [159]. Whether the altered exosomal communication is involved in the progression of hypertension-associated neurodegenerative diseases is largely unknown. Additionally, proteins including CD36, flotillin-1, alpha-sarcoglycan, HSP72, and Aβ have been observed in exercise-induced EVs [160]. However, the biological functions of these proteins are currently unclear.

EVs can also serve as vehicles to carry therapeutic molecules to treat cognitive decline. Proteins such as catalase can be packaged into EXs derived from macrophages and monocytes, which can be taken up by neurons to reduce oxidative stress in PD [161]. Studies have also found that stimulating astrocytes via inflammation and oxidative stress may induce them to release EVs, which can promote recovery and might be used to regenerate cells post-dementia injury [105]. Other research groups also loaded glial-cell-derived neurotrophic factors into EXs and introduced them to a monkey PD model. They found that these engineered EXs exhibited a strong neuroprotective effect [162]. All these findings indicate that EXs are promising carriers for delivering drugs to the central nervous system, but more research is needed in the future on the delivery of therapeutic drugs such as siRNAs and miRNAs and on the development of instruments or methods for engineering EXs.

## 4. Summary, Limitations, and Future Directions

The field of EV research is a rapidly developing field. Over the past few decades, EVs have evolved from a means to remove toxic or unwanted molecular materials for maintaining cell homeostasis into a key player in various biological processes. EVs have reduced immunogenicity due to their biocompatibility and a bi-layered lipid structure, which protects the genetic cargo from degradation, making them attractive as drug delivery vehicles. The nanosize and membrane composition of EVs allows them to cross the biological membranes including the BBB. In the central nervous system, EVs are shown to harbor pathogenic proteins such as synuclein and tau proteins, lipids, and genetic materials and exhibit numerous functions. An increasing number of preclinical and clinical studies reveal that EVs are implicated in the pathogenesis, diagnosis, and therapeutics of neurodegenerative diseases. In addition to contributing to the pathogenesis of neurodegenerative diseases, EVs have the promise of serving as biomarkers for such neural complications.

EVs can be secreted from diseased cells but also non-diseased cells in the brain. Hence, EV isolation (heterogeneity of subpopulations) and cargoes (active biomolecules) are tremendously important for strengthening diagnostic interpretations. Therefore, standardizing the isolation, purification, and storage methods and elucidating the precise mechanisms governing the sorting and integration of biological material into EVs are urgently needed. However, to date, there is a lack of an optimal, efficient, and reliable isolation method for the clinical applications of EVs. Several techniques have been developed for isolating EVs, including ultracentrifugation, density-gradient centrifugation, ultrafiltration, immunoaffinity chromatography, the microfluidic chip-based method, and commercial EX isolation kits such as ExoQuick or ExoQuick-Ultra. However, each of these methods has its pros and cons. For example, immunoaffinity-based techniques require expensive antibodies. The microfluidic technique shows great promise, but it requires complex chip fabrication. In future studies, advanced technologies are also required to minimize contaminants, particularly in the plasma and serum, and to clearly validate the key biological and pathological components in EVs. Meanwhile, it remains challenging to isolate circulating EVs derived from the brain and identify specific types of brain cells [26]. Discovering more specific markers and developing more innovative separation methodologies are urgently needed. Notably, there is a great progress of the EV sensing techniques, including electrochemical sensors [163,164,165], plasmonic-based sensors [166,167], and the polymer Bragg grating-based refractive index sensor [168]. Such techniques aim to improve the sensitivity of quantifying EVs and enhance the application of EVs in translational research including neurodegenerative diseases, cardiovascular diseases, and cancers.

EV uptake mechanisms and targeting properties are important for regulating the recipient cellular functions. Regarding the EV internalization pathways, several mechanisms have been proposed, including membrane fusion, clathrin-mediated endocytosis, caveolin-mediated endocytosis, lipid raft-mediated endocytosis, micropinocytosis, or phagocytosis. The uptake mechanism varies depending on the presence of various receptors on both EVs and intended cell targets [10,169,170]. It is unclear whether EVs target cells in a nondiscriminatory manner or if they utilize a preferential system [171,172,173]. Therefore, in future studies, elucidating EV uptake mechanisms, identifying EV homing, and targeting the specificities of EVs of different cellular origins are needed to match the application potential of EVs for clinical use.

Additionally, engineering bioactive EXs is an active research field, which fosters the assessment of various therapeutic cargoes, enhancement of target selectivity, and optimization of manufacturing for neurodegenerative diseases. However, a major concern for the successful translation of EVs is to precisely target the cell type of interest while limiting off-target biodistribution in the brain. Another concern is the presence of naturally incorporated cellular genetic impurities with potential immunogenicity. To circumvent these difficulties, a better understanding of EX biology to improve therapeutic EX engineering is necessary.

Taken together, an increasing number of studies support the involvement of EVs in neurodegenerative diseases. However, the specific roles of EVs in the context of neurodegenerative diseases are still not well understood. An improved understanding of the biology of EV biogenesis, contents, targeting, and uptake mechanisms by target cells, as well as identifying the subpopulations of EVs and their specific cargo, would also facilitate the utilization of EVs for novel diagnostics and prognostics in neurodegenerative diseases.

## Figures and Tables

**Table 2:** Findings on the roles of EVs in the pathogenicity and/or diagnosis of AD and PD.

EV Source	Contents	Involvement of EV in AD and PD Progression/Diagnosis	Reference
Endothelial cells	Aβ	Carry toxic proteins across the BBB.	[95]
Brain-derived EVs	AD	Carry pathological biomaterials (Aβ and tau) and glial-specific molecules (ANXA5, VGF, GPM6A, and ACTZ)	[118]
Neuron	Aβ	Spread between neurons in a prion-like manner, or from neurons to microglia.	[123,130]
	Aβ and tau proteins, and miRNA/mRNA	Biomarker sourced from blood/plasma samples.	[128,129,130,132]
	Toxic tau proteins	Biomarker derived from CSF samples	[126]
Astrocytes	Aβ	Trafficked from astrocytes to neurons	[127]
	Inflammatory proteins	Biomarker from blood/plasma samples	[131]
	Aβ and tau proteins	Biomarker from blood/plasma samples	[105,127,128]
Microglia	Aβ	Distribute to other microglial or neuron cells.	[115,122,130]
	Tau	Contribute to the progression of tauopathy by spreading tau to other brain cells.	[120]
	miRNA	Biomarker	[135]

## Data Availability

Not applicable.
